# Visualizing the Achilles Tendon Enthesis: Translational Insights from 7-Tesla MR Microscopy and Histology

**DOI:** 10.3390/diagnostics16091257

**Published:** 2026-04-22

**Authors:** Johannes M. Mittendorfer, Zehra Duezguen, Elisabeth M. Mandler, Henning Tewes, Martin Zalaudek, Lena Hirtler

**Affiliations:** 1 Division of Anatomy, Center for Anatomy and Cell Biology, Medical University of Vienna, 1090 Vienna, Austria; johannes.mittendorfer@meduniwien.ac.at (J.M.M.); zehra.duezguen@meduniwien.ac.at (Z.D.); elisabeth.mandler@meduniwien.ac.at (E.M.M.);; 2 Department for Radiology and Image Guided Therapy, Medical University of Vienna, 1090 Vienna, Austria

**Keywords:** Achilles tendon, calcaneal tendon, enthesis, musculoskeletal disorders, magnetic resonance imaging, 7-Tesla MRI, MR microscopy, histology, osteotendinous junction

## Abstract

**Background/Objectives:** The Achilles tendon enthesis (ATE) is a key load-transmitting structure that is frequently affected in musculoskeletal disorders, including insertional tendinopathy, overuse injuries and inflammatory enthesopathies. Reliable non-invasive assessment of the enthesis structure is therefore of increasing clinical importance. This study evaluated the ability of advanced magnetic resonance (MR) microscopy to depict the ultrastructural organization of the ATE using histology as a reference standard. **Methods:** Five human ATEs from anatomical body donations were included. Two specimens were used for protocol development of the histological preparation, whereas three specimens underwent the full multimodal pipeline comprising undecalcified methyl methacrylate (MMA) thin-section histology with Giemsa staining, T2*-weighted 3D-variable echo time (vTE) MR microscopy at 7 Tesla, and microradiography. **Results:** Histological analysis demonstrated excellent preservation of fibrocartilage zones and mineralized interfaces. Corresponding MR microscopy data allowed the identification of major structural components of the enthesis, particularly mineralized regions, although fine ultrastructural details remained beyond the MR microscopy resolution. Microradiography supported interpretation of the mineralized tissue architecture and MR microscopy signal characteristics. **Conclusions:** These findings indicate that high-field MR microscopy can capture clinically relevant structural features of the Achilles tendon enthesis, while histology remains essential for detailed ultrastructural validation. The combined imaging approach provides a translational framework that may support improved diagnosis, monitoring and treatment evaluation in musculoskeletal disorders involving the osteotendinous junction.

## 1. Introduction

The Achilles tendon is the strongest and thickest tendon in the human body, capable of transmitting forces exceeding ten times body weight during activities such as running and jumping [1,2]. Functionally, it connects the triceps surae muscle complex to the calcaneus and plays a critical role in plantarflexion and energy storage during gait [2,3]. The site of insertion into the posterior calcaneus, termed the Achilles tendon enthesis (ATE), represents a highly specialized osteotendinous junction adapted to withstand substantial mechanical loading [4,5,6].

The ATE is classified as a fibrocartilaginous enthesis, characterized by a gradual transition from dense collagenous tendon through non-mineralized and mineralized fibrocartilage to subchondral bone [4,5,6,7]. This zonal architecture facilitates stress dissipation and minimizes stress concentration at the tendon–bone interface [5,8]. In addition to the enthesis itself, the region forms part of a functional “enthesis organ” that includes adjacent structures such as periosteal fibrocartilage, the retrocalcaneal bursa, and Kager’s fat pad, which together contribute to load distribution and friction reduction during movement [9,10,11].

Owing to its complex biomechanical environment, the ATE is frequently involved in both degenerative and inflammatory pathologies. Degenerative changes are commonly observed in ageing populations and athletes exposed to repetitive mechanical strain [12], whereas inflammatory involvement of the enthesis is a hallmark of spondyloarthropathies [9,13]. Structural alterations at the fibrocartilaginous interface are increasingly recognized as being central to the pathogenesis of enthesopathies, making early detection of ultrastructural changes clinically relevant for diagnosis, disease monitoring and therapeutic decision-making [9,13,14].

Magnetic resonance imaging (MRI) is regarded as the reference standard for non-invasive assessment of soft tissues and is widely used for the evaluation of Achilles tendon disorders [15,16]. However, conventional MRI sequences are limited in their ability to visualize mineralized fibrocartilage and calcified tissue components due to their extremely short T2 and T2* relaxation times [15,17]. Advances in MRI technology, including ultrashort echo time (UTE) and variable echo time (vTE) techniques, have improved sensitivity to short-T2 tissues and enabled partial visualization of enthesis mineralization [15,18,19]. Despite these developments, the spatial resolution and signal contrast remain insufficient to resolve fine ultrastructural features in vivo, necessitating in vitro MR approaches and histological validation [7,15].

Histological analysis remains the gold standard for detailed evaluation of the enthesis microarchitecture. The undecalcified methyl methacrylate (MMA) thin-section technique according to Plenk allows simultaneous visualization of mineralized and non-mineralized tissues without decalcification, preserving both the cellular morphology and extracellular matrix organization. When combined with appropriate staining protocols, such as Giemsa, this technique provides high-resolution insight into fibrocartilage zones, tidemarks, and the osteotendinous interface [10]. Nevertheless, histological methods are inherently ex vivo and subject to preparation-related artifacts, limiting their direct clinical applicability.

To address the gap between non-invasive imaging and ultrastructural tissue characterization, comparative approaches integrating advanced MRI and histology are required. High-field MRI at 7 Tesla has demonstrated potential for musculoskeletal applications, including tendon and cartilage imaging, by offering improved signal-to-noise ratio and spatial resolution [15,19,20]. However, studies focusing specifically on the Achilles tendon enthesis and systematically correlating MRI findings with histological workup remain scarce [8].

The aim of this study was threefold: first, to establish an ex vivo multimodal workflow for the Achilles tendon enthesis using 7-Tesla 3D-vTE MR microscopy, microradiography, and undecalcified MMA thin-section histology; second, to assess whether MR microscopy can depict the major structural zones of the enthesis in comparison with histology and microradiography; and third, to define the specific strengths and limitations of each modality for future translational application.

## 2. Materials and Methods

### 2.1. Specimen Collection and Preparation

This anatomical study was conducted on five Achilles tendon entheses (ATEs) obtained from fresh lower legs originating from voluntary human body donors to the Centre for Anatomy and Cell Biology, Medical University of Vienna, Austria. The five donors (three female, two male) had a mean age of 80.8 years (range 77–93 years). All specimens were free of visible pathologies and had no documented history of surgical interventions or trauma to the Achilles tendon region. Two of the five specimens were designated for protocol development and optimization of the histological procedures, while three were used for the full experimental pipeline, including imaging, histology and microradiography.

Each specimen was dissected to isolate the calcaneus with the attached Achilles tendon, preserving the natural orientation of the enthesis. Care was taken to maintain the anatomical integrity of the surrounding soft tissue structures, including Kager’s fat pad and adjacent bursal tissues, to ensure anatomical correlation during imaging and sectioning.

### 2.2. Regions of Interest (ROIs)

Because of the anatomical complexity of the Achilles tendon enthesis, morphological analysis was focused on three predefined regions of interest (ROIs). ROI 1 comprised the osteotendinous junction, including the characteristic four zones of a fibrocartilaginous enthesis. ROI 2 encompassed the retrocalcaneal components of the enthesis organ, with particular focus on Kager’s fat pad and the retrocalcaneal bursa with its fibrocartilaginous attachments. ROI 3 represented the distal Achilles tendon immediately proximal to the insertion, defined as the tendon segment between two imaginary parallel perpendicular lines drawn below the retrocalcaneal bursa and through the most distal identifiable point of the osteotendinous junction.

### 2.3. Magnetic Resonance Microscopy Protocol

MRI was performed on three of the five specimens after resection. For imaging, the Achilles tendon entheses were fixed in plastic syringes with a diameter of 35 mm, filled with 0.9% saline solution and sealed airtight. The syringes were placed in a 39 mm 1H-NMR volume resonator (Rapid Biomedical, Würzburg, Germany). Measurements were performed on a 7-Tesla ultrahigh-field MRI system (Siemens Healthineers, Erlangen, Germany) using a microgradient system with a maximum gradient strength of 750 mT/m and 3D-vTE sequence with integrated water-selective binomial 1-1 excitation for fat suppression. A total of 12 echo times were acquired: 0.45, 1.25, 2.05, 2.85, 4.42, 5.42, 6.42, 7.42, 8.24, 9.24, 10.24 and 11.24 ms. Additional acquisition parameters were as follows: field of view = 32.4 × 58 mm^2^; pixel size = 0.15 × 0.15 mm^2^; slice thickness = 0.15 mm; number of slices = 256; flip angle = 15°; repetition time = 15 ms; acquisition time = 70 min 31 s.

Morphological evaluation of the sagittal images was performed using JiveX[dv]Viewer (VISUS Health IT GmbH, Bochum, Germany). For each specimen, the image series with the most suitable contrast for morphological assessment was selected first. The central sagittal slice plane of the enthesis was then identified based on the total number of slices. The evaluation focused on the three predefined regions of interest. After initial inspection of the two-dimensional image, the regions of interest were further assessed throughout the image stack, while the remaining image series acquired at different echo times were used for comparisons of tissue contrast in individual structures.

### 2.4. Microradiography (MRG)

To further support the morphological evaluation, microradiographic imaging was conducted on the same sections used for histology. The sections were scanned using a cabinet X-ray system (Hewlett Packard CO. Cabinet X-Ray System—Faxitron Series, McMinnville, OR, USA, 1987) with a resolution of 10 µm. This method provides high-resolution images of mineralized structures and is particularly effective for visualizing trabecular bone, tidemarks and mineralized fibrocartilage.

MRG images served as an intermediate modality, bridging the resolution gap between MRI and histological slices. They were used to assess the continuity of mineralization zones and to validate signal voids or artifacts observed in MRI data.

### 2.5. Histological Technique: Undecalcified MMA Thin-Section Method (Plenk Technique)

The central methodological approach employed in this study was the undecalcified MMA thin-section technique according to Plenk [21], which enables high-resolution visualization of mineralized and non-mineralized tissues without decalcification. The entheses were dehydrated through a graded ethanol series and embedded in MMA. Subsequently, median sagittal sections of approximately 100–150 µm thickness were obtained using a saw microtome for direct comparison with MR microscopy. These sections were mounted onto glass slides and stained with Giemsa, which provides strong contrast for cellular components, extracellular matrix and calcified tissues.

A key advantage of this technique lies in its ability to preserve mineralized fibrocartilage, tidemarks and the calcified interface between tendon and bone. The resulting sections were examined under a light microscope at various magnifications (x40 to x400) and digitally scanned to allow for quantitative and qualitative comparisons with imaging modalities.

### 2.6. Image Analysis and Correlation

Histological sections, MR images, and microradiographs were analyzed in parallel. The analysis was performed by two raters (H.T. and L.H.) reaching a consensus. Regions of interest (ROIs) were defined within the mineralized fibrocartilage, tidemark and adjacent bone. The qualitative analysis focused on structural integrity, continuity and tissue contrast. Quantitative parameters, such as the fibrocartilage thickness and enthesis angle, were also recorded where feasible.

Special attention was paid to the correlation of MRI signal behavior at different echo times with specific histological features, particularly in the transition zones between tendon, fibrocartilage and bone. This multimodal approach enabled a robust assessment of the strengths and limitations of each modality.

## 3. Results

### 3.1. Histological Observations

The Giemsa-stained undecalcified MMA thin sections revealed well-preserved tissue architecture across all examined specimens. The transition from dense collagenous tendon to non-mineralized and mineralized fibrocartilage was clearly distinguishable. Mineralized fibrocartilage appeared as a basophilic zone with reduced cellularity and a homogenous matrix structure, followed by sharply defined tidemarks separating it from the underlying trabecular bone. The length of the Achilles tendon enthesis in undecalcified thin sections was 2.03 ± 0.42 cm (median 1.92 cm; range 1.56–2.56 cm).

Characteristic features of fibrocartilaginous entheses, including columnar chondrocyte alignment, interdigitating interfaces and anisotropic matrix patterns, were observed with excellent clarity. Additionally, adjacent structures such as the Kager’s fat pad and retrocalcaneal bursa were identifiable in situ, aiding anatomical orientation. Minor degenerative features, such as fragmented tidemarks and reduced cellularity in the elderly donor tissue, were occasionally observed but did not impair section quality.

**Figure 1:**
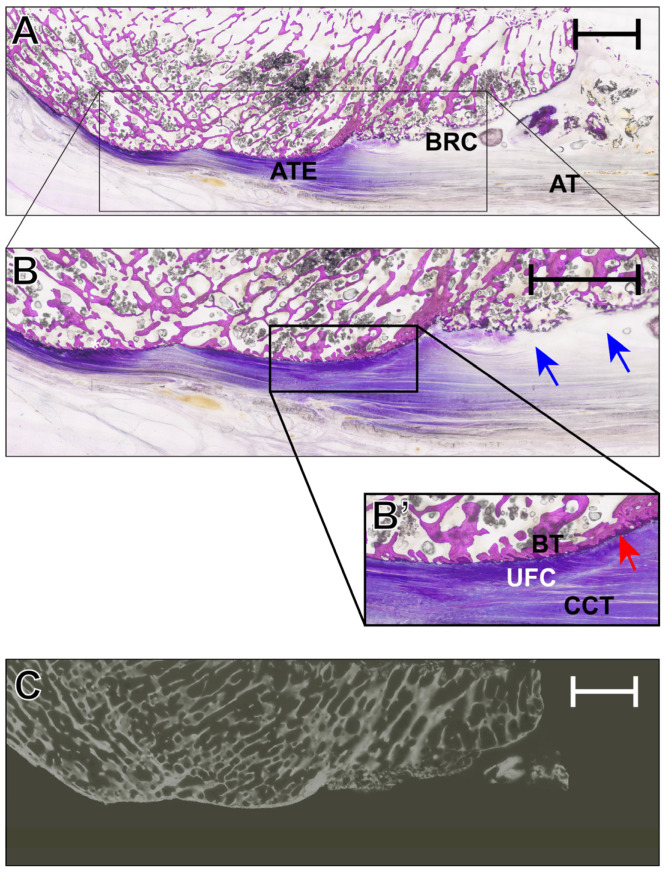
(**A**) Histological overview of specimen IV in the median sagittal section after undecalcified Giemsa-stained thin-ground-section preparation of the Achilles tendon enthesis (ATE). (**B**) Thin-ground section showing the calcaneal bone block, Achilles tendon enthesis, retrocalcaneal bursa, and distal Achilles tendon (AT). The trabecular bone appears overall rarefied, and the dorsal peritendinous soft tissue is preserved. (**B’**) Enlarged view of the enthesis demonstrating the zonal transition of the insertion (BT = bone tissue; UFC = unmineralized fibrocartilage; CCT = collagenous connective tissue), a multiloculated retrocalcaneal bursa (RCB, blue arrows), focal cortical discontinuity, and intrabursal tissue fragments or calcified material as well as the basophile tidemark (red arrow). (**C**) Corresponding microradiography. The scale bar reflects 5 mm.

The histological assessment of specimen IV as seen in Figure 1 revealed a preserved median sagittal section of the Achilles tendon enthesis with clear delineation of the calcaneal bone block, enthesis, retrocalcaneal bursa, and distal Achilles tendon. The spongiosa appeared markedly reduced in density, whereas the dorsal peritendinous soft tissue remained largely intact. Higher magnification confirmed the zonal organization of the enthesis and demonstrated a multiloculated retrocalcaneal bursa, with fibrocartilaginous components confined to its inferior aspect. Moreover, focal cortical interruption and intrabursal tissue fragments or calcified material were noted in the proximal insertional region.

### 3.2. MR Microscopy Signal Characteristics

MRI scans at 7 Tesla demonstrated good visualization of the gross anatomy of the Achilles enthesis. With shorter echo times (1.3–4.8 ms), high signal intensity was observed in the unmineralized tendon and fibrocartilage regions, while mineralized zones exhibited rapid signal decay, resulting in signal voids. As the echo time increased, the differentiation between mineralized and non-mineralized regions improved, but fine structural details such as the tidemark remained incompletely resolved.

For ROI 2, the retrocalcaneal region could be identified morphologically, with variable depiction of the bursal contour and surrounding soft tissue interfaces depending on the echo time. For ROI 3, the distal Achilles tendon showed progressive TE-dependent signal loss with improved visualization of the internal fascicular architecture at longer echo times.

Despite these limitations, the overall correlation between histological mineralization patterns and MRI signal profiles was consistent. The 3D-vTE sequence proved effective in differentiating between soft tissue and hard tissue zones, although it lacked the resolution to distinguish microstructural features such as tidemark integrity or cellular arrangement.

**Figure 2:**
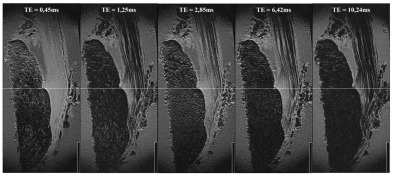
Representative sagittal 3D-vTE MRI images of specimen IV acquired at five different echo times (TE = 0.45, 1.25, 2.85, 6.42, and 10.24 ms). The image series demonstrates TE-dependent signal changes within the Achilles tendon enthesis region. With increasing TE, the collagenous tendon shows progressive signal loss, resulting in improved delineation of the fascicular architecture. Cortical bone remains persistently hypointense throughout the sequence, while trabecular organization in the distal calcaneus is most appreciable at very short TE. The scale bar corresponds to 1 cm.

Figure 2 illustrates the influence of echo time on tissue contrast within the Achilles tendon enthesis. With increasing TE, progressive signal loss is observed in the collagenous tendon, resulting in clearer delineation of the fascicular architecture, whereas the cortical bone remains consistently hypointense and is best distinguished at shorter-to-intermediate TE values.

### 3.3. Microradiography Comparison

**Figure 3:**
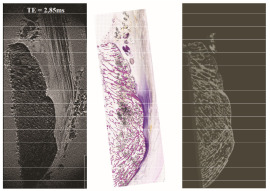
Direct comparison of the MRI (TE = 2.85 ms) and microradiography of specimen IV. Note the difference in accuracy of the depiction of the trabecular structure of the calcaneal bone and the depiction of the tidemarks in the MRI. The scale bar corresponds to 1 cm.

**Figure 4:**
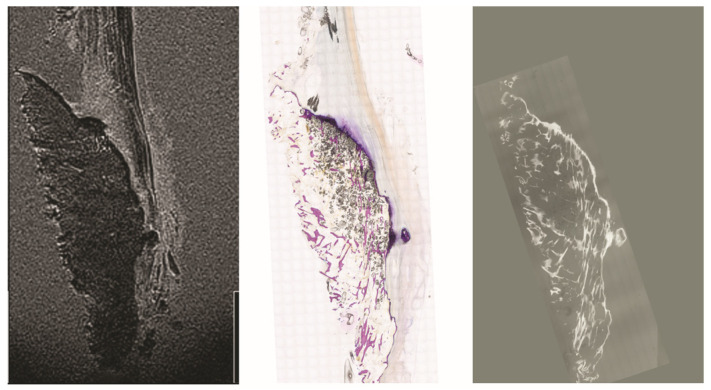
Direct comparison of the MRI (TE = 2.85 ms) and microradiography of specimen V. Note the diminished trabecular structure of the calcaneal bone in the microscopical image and the microradiography also in comparison with Figure 3. The scale bar corresponds to 1 cm.

Microradiographs of the same sections used for histology provided excellent detail of the mineralized matrix. The contrast among trabecular bone, mineralized fibrocartilage, and tidemark zones was markedly sharper than that in MRI (see Figure 3 and Figure 4). Notably, the interface between bone and fibrocartilage exhibited an irregular, interdigitated pattern in all samples. These findings closely matched the mineralized patterns observed in histology.

When aligned with MRI images, the microradiographs helped interpret signal voids and clarified whether these corresponded to genuine mineralization or technical artifacts. They also confirmed the relative thickness and topography of the mineralized fibrocartilage zone across samples.

As expected, microradiography was most informative for mineralized components and contributed less to direct assessment of the non-mineralized distal tendon, but it improved interpretation of the insertional topography adjacent to ROI 2 and ROI 3.

### 3.4. Modality Comparison

The combination of MRI, microradiography and histology yielded a comprehensive overview of the Achilles tendon enthesis architecture. Histology provided unmatched resolution and structural specificity, allowing clear visualization of cellular and matrix features. Microradiography bridged the resolution gap, particularly in the mineralized interface, while MR microscopy offered the only clinically translatable imaging technique with potential for in vivo application. Limitations were evident in MRI’s inability to resolve fine ultrastructural components such as individual chondrocyte columns or early degenerative changes in fibrocartilage.

## 4. Discussion

This study provides an in-depth morphological and imaging-based evaluation of the human Achilles tendon enthesis (ATE), with particular focus on the capacity of advanced imaging modalities to reflect its ultrastructural features. The combination of high-resolution histology using the undecalcified MMA thin-section technique according to Plenk [22], high-resolution T2*-weighted 3D-variable echo time (vTE) magnetic resonance imaging (MRI) at 7 Tesla, and microradiography (MRG) enabled a comprehensive multimodal assessment of the enthesis region. Only limited studies in the literature have addressed direct Achilles enthesis validation using advanced MR microscopy in conjunction with histological reference techniques [23].

The Achilles tendon enthesis is a fibrocartilaginous osteotendinous junction, which features a gradual transition from dense tendon to mineralized fibrocartilage and ultimately to trabecular bone [4,5,6,7,24]. This architectural complexity facilitates efficient load transmission while mitigating mechanical stress concentration [5,7,24]. Histological analysis confirmed the expected zonal architecture with clearly defined tidemarks, mineralized fibrocartilage, and adjacent trabecular bone. These findings were consistent across all specimens, suggesting that the undecalcified MMA thin-section technique reliably preserves enthesis integrity, even in elderly donor tissue.

The 7T MRI data acquired using the 3D-vTE sequence demonstrated good anatomical detail in the non-mineralized components of the enthesis, particularly within the tendon and unmineralized fibrocartilage [25]. However, the mineralized zone exhibited signal voids due to rapid transverse relaxation and susceptibility effects inherent to hard tissues [15,17]. While the use of multiple echo times improved tissue contrast and the delineation of gross anatomical boundaries, the MRI resolution remained insufficient to detect cellular-level features such as chondrocyte columns, tidemarks, or early microstructural degeneration [23].

These limitations highlight the continuing gap between non-invasive imaging modalities and ex vivo histological methods. Although MRI remains indispensable for in vivo clinical assessment, its current technical parameters—particularly in musculoskeletal applications—do not yet allow for true replication of histological detail at the osteotendinous interface [23]. Still, the promising tissue differentiation achieved with 3D-vTE suggests that with further refinement, particularly in coil technology and sequence design, MRI may gain improved sensitivity for fibrocartilaginous insertions [26].

Microradiography served as an essential intermediate tool in this study, particularly for its ability to reveal mineralized tissue architecture. The high contrast and spatial resolution achieved through microradiography allowed clear visualization of trabecular bone and mineralized fibrocartilage, which closely matched corresponding histological patterns. Furthermore, MRG helped clarify ambiguous MRI findings by confirming whether observed signal voids represented true mineralization or imaging artifacts. Its value lies not only in its imaging capabilities but also in bridging the interpretive gap between in vivo-compatible MRI and high-resolution ex vivo histology.

An important observation in this study is that MRI and histology provide complementary, rather than competing, perspectives. While histology offers unmatched structural clarity, it is inherently invasive and unsuitable for serial monitoring or patient-specific diagnostics. MRI, by contrast, is safe, non-invasive, and already integrated into clinical workflows. The strength of this study lies in its side-by-side evaluation of both techniques, which can be used to calibrate MRI interpretation and validate future clinical protocols targeting the enthesis [26].

From a translational perspective, this approach holds significant relevance for the diagnosis and monitoring of enthesopathies. Conditions such as enthesitis in spondyloarthropathies, chronic tendinopathies, or insertional Achilles tendinitis involve microstructural changes at the fibrocartilaginous interface [27]. The ability to detect early enthesis changes through advanced MRI protocols could facilitate earlier interventions, aid in monitoring therapy effectiveness, and ultimately reduce chronic degeneration or rupture risk [28].

Nonetheless, several limitations must be acknowledged. First, all specimens were from elderly body donors with an average age over 80 years. Age-related changes such as decreased cellularity, reduced fibrocartilage thickness, and tidemark irregularities may not reflect the enthesis morphology of younger or athletic populations [24]. Secondly, tissue fixation, dehydration, and embedding steps in histology can introduce artifacts that alter natural tissue dimensions, despite best efforts to standardize protocols. Third, the MRI imaging in this study was performed ex vivo. In vivo MRI at 7T remains limited to specialized research centers and is not yet standard in clinical musculoskeletal practice.

A further limitation regarding clinical translation is the long acquisition time of 70 min 31 s used in the present ex vivo protocol, which is not compatible with routine in vivo musculoskeletal imaging and therefore underscores the exploratory, proof-of-concept character of this study. Another important limitation regarding translation is the advanced age of the donor cohort. The specimens were derived from body donors with a mean age of 80.8 years, which reflects the characteristics of the available anatomical material rather than a representative clinical population. Age-related changes in fibrocartilage, mineralization, cellularity, and tendon structure may have influenced both the histological findings and the MR microscopy appearance and, therefore, limit direct extrapolation to younger, athletic, or symptomatic patient cohorts.

Furthermore, our study did not perform quantitative morphometry or signal intensity analysis beyond descriptive comparison. Future work could expand upon this by implementing quantitative image analysis, automated segmentation, and correlation with biomechanical or clinical data. Additionally, applying this methodology to pathological specimens—such as those with confirmed enthesitis or tendinopathy—could further validate the clinical utility of advanced MRI sequences in detecting disease-relevant features [27,28].

In summary, this study highlights the strengths and weaknesses of both MRI and histological approaches in characterizing the Achilles tendon enthesis. While histology remains the gold standard for detailed ultrastructural assessment, high-resolution MRI at 7 Tesla using 3D-vTE sequences shows promise for identifying key morphological zones and may eventually support non-invasive diagnostics in enthesis-related pathologies [25,27]. Combining imaging and histology within a multimodal framework offers a powerful strategy for both basic science and translational research on musculoskeletal insertional structures.

## 5. Conclusions

This study demonstrated the value of a multimodal approach to analyzing the Achilles tendon enthesis by integrating high-resolution histology, microradiography, and high-resolution 7-Tesla MRI using a T2*-weighted 3D-variable echo time sequence. The undecalcified MMA thin-section technique according to Plenk provided detailed visualization of the entheses’ ultrastructural composition, including the mineralized fibrocartilage and tidemark. Microradiography confirmed mineralization patterns and enhanced the interpretation of MRI data. While MRI offered non-invasive access to soft tissue structures and broad architectural zones, it lacked the resolution to capture the fine ultrastructural details observed in histological sections.

The findings confirm that MR microscopy and histology are complementary modalities: histology remains indispensable for cellular and matrix-level evaluation, while MRI presents a promising in vivo-compatible tool that could, with further optimization, support early diagnosis of enthesis-related disorders. The results encourage the continued refinement of imaging techniques and support the development of hybrid diagnostic strategies in musculoskeletal medicine.

This work establishes a methodological foundation for future research focused on the enthesis, particularly in degenerative or inflammatory conditions. Bridging the gap between clinical imaging and microscopic anatomy offers meaningful opportunities to improve patient outcomes through earlier detection, personalized therapy planning, and targeted monitoring.

## Data Availability

The original contributions presented in this study are included in the article. Further inquiries can be directed to the corresponding author.

## Abbreviations

The following abbreviations are used in this manuscript:

ATE	Achilles tendon enthesis
MMA	Methyl methacrylate
MR	Magnetic resonance
MRI	Magnetic resonance imaging
ROI	Region of interest
UTE	Ultrashort echo time
vTE	Variable echo timeTE Echo time

## References

[1] Benjamin M., Kaiser E., Milz S. (2008). Structure-function relationships in tendons: A review. J. Anat..

[2] Maganaris C.N., Narici M.V., Maffulli N. (2008). Biomechanics of the Achilles tendon. Disabil. Rehabil..

[3] O’Brien M. (2005). The anatomy of the Achilles tendon. Foot Ankle Clin..

[4] Benjamin M. (2002). The skeletal attachment of tendons–tendon “entheses”. Comp. Biochem. Physiol. Part A Mol. Integr. Physiol..

[5] Benjamin M. (2006). Where tendons and ligaments meet bone: Attachment sites (‘entheses’) in relation to exercise and/or mechanical load. J. Anat..

[6] Burgkart R. (2026). Tendon-bone interface—Nature’s solution for a hard-soft-interface. Ann. Anat..

[7] Benjamin M., Ralphs J.R. (1998). Fibrocartilage in tendons and ligaments—An adaptation to compressive load. J. Anat..

[8] Rossetti L., Kuntz L.A., Kunold E., Schock J., Muller K.W., Grabmayr H., Stolberg-Stolberg J., Pfeiffer F., Sieber S.A., Burgkart R. (2017). The microstructure and micromechanics of the tendon-bone insertion. Nat. Mater..

[9] Benjamin M., McGonagle D. (2009). The enthesis organ concept and its relevance to the spondyloarthropathies. Adv. Exp. Med. Biol..

[10] Milz S., Benjamin M., Boszczyk A.A., Putz R. (2005). Die Enthesis—Physiologische Morphologie, molekulare Zusammensetzung und pathoanatomische Veränderungen. Orthopäde.

[11] Shaw H.M., Benjamin M., Kaiser E., Milz S., Ralphs J.R. (2008). Development of the human Achilles tendon enthesis organ. J. Anat..

[12] Paavola M. (2002). Achilles tendinopathy. J. Bone Jt. Surg. Am..

[13] Benjamin M., McGonagle D. (2001). The anatomical basis for disease localisation in seronegative spondyloarthropathy at entheses and related sites. J. Anat..

[14] Benjamin M., McGonagle D. (2009). Entheses: Tendon and ligament attachment sites. Scand. J. Med. Sci. Sports.

[15] Benjamin M., Milz S., Bydder G.M. (2008). Magnetic resonance imaging of entheses. Part 1. Clin. Radiol..

[16] Pierre-Jerome C., Moncayo V., Terk M.R. (2010). MRI of the Achilles tendon: A comprehensive review of the anatomy, biomechanics, and imaging of overuse tendinopathies. Acta Radiol..

[17] Chavhan G.B., Babyn P.S., Thomas B., Shroff M.M., Haacke E.M. (2009). Principles, techniques, and applications of T2*-based MR imaging and its special applications. Radiographics.

[18] Robson M.D., Gatehouse P.D., Bydder M., Bydder G.M. (2003). Magnetic resonance: An introduction to ultrashort TE (UTE) imaging. J. Comput. Assist. Tomogr..

[19] Crombé A., Dallaudière B., Bohand M.C., Fournier C., Spinnato P., Poursac N., Carl M., Poujol J., Hauger O. (2025). Zero and Ultra-Short Echo Time Sequences at 3-Tesla Can Accurately Depicts the Normal Anatomy of the Human Achilles Tendon Enthesis Organ In Vivo. J. Clin. Med..

[20] Hager B., Walzer S.M., Deligianni X., Bieri O., Berg A., Schreiner M.M., Zalaudek M., Windhager R., Trattnig S., Juras V. (2018). Orientation dependence and decay characteristics of T2* relaxation in the human meniscus studied with 7 Tesla MR microscopy and compared to histology. Magn. Reson. Med..

[21] Romeis B. (1989). Mikroskopische Technik..

[22] Deligianni X., Wurnig M.C., Boss A. (2013). High-resolution Fourier-encoded sub-millisecond echo time musculoskeletal imaging at 3 Tesla and 7 Tesla. Magn. Reson. Med..

[23] Juras V., Apprich S., Pressl C., Zbyn S., Szomolanyi P., Domayer S., Hofstaetter J.G., Trattnig S. (2013). Histological correlation of 7 T multi-parametric MRI performed in ex-vivo Achilles tendon. Eur. J. Radiol..

[24] Apostolakos J., Durant T.J., Dwyer C.R., Russell R.P., Weinreb J.H., Alaee F., Beitzel K., McCarthy M.B., Cote M.P., Mazzocca A.D. (2014). The enthesis: A review of the tendon-to-bone insertion. Muscles Ligaments Tendons J..

[25] Han M., Larson P.E., Liu J., Krug R. (2014). Depiction of achilles tendon microstructure in vivo using high-resolution 3-dimensional ultrashort echo-time magnetic resonance imaging at 7 T. Investig. Radiol..

[26] Deligianni X., Wurnig M.C., Trattnig S., Boss A. (2014). Water-selective excitation of short T2 species with binomial pulses. Magn. Reson. Med..

[27] Chen B., Zhao J., Zeng M., Wu Y., Chang E.Y., Du J. (2018). Three-dimensional ultrashort echo time cones (3D UTE-Cones) magnetic resonance imaging of entheses and tendons. Magn. Reson. Imaging.

[28] Moazamian D., Athertya J.S., Dwek S., Lombardi A.F., Mohammadi H.S., Sedaghat S., Jang H., Ma Y., Chung C.B., Du J. (2024). Achilles tendon and enthesis assessment using ultrashort echo time magnetic resonance imaging (UTE-MRI) T1 and magnetization transfer (MT) modeling in psoriatic arthritis. NMR Biomed..

